# Pathological Complete Response After Neoadjuvant Imatinib in a Recurrent Duodenal Gastrointestinal Stromal Tumor (GIST)

**DOI:** 10.7759/cureus.64669

**Published:** 2024-07-16

**Authors:** Joana Marques-Antunes, Lucia Carvalho, Silvia Pereira, Tiago Ferreira, Mário Nora

**Affiliations:** 1 General Surgery, Centro Hospitalar de Entre o Douro e Vouga, Santa Maria da Feira, PRT

**Keywords:** surgical procedures, locoregional neoplasm recurrence, pathological complete response (pcr), imatinib therapy, gastrointestinal stromal tumor (gist), neoadjuvant therapy

## Abstract

Gastrointestinal stromal tumors (GISTs) are the most frequent mesenchymal neoplasms of the gastrointestinal (GI) tract. Although surgery is the treatment of choice in resectable disease, neoadjuvant therapy is indicated in advanced, metastatic, and recurrent tumors. Decreasing tumor burden may facilitate resection and reduce surgical morbidity. We describe a case of a 66-year-old male with a recurrent duodenal GIST, after surgery and adjuvant imatinib five years before. Following neoadjuvant therapy with imatinib for 12 months, the patient underwent a cephalic pancreaticoduodenectomy, without complications. The final histopathology showed a pathological complete response (pCR) with no residual neoplasm. A pathological complete response to imatinib in a recurrent disease is extremely rare. Molecular testing should be performed before neoadjuvant therapy to identify response-predictive mutations. In recurrent/metastatic disease, systemic therapy is the standard treatment for all patients. Surgery should be considered in a tailored approach in patients with good responses to systemic therapy before developing therapeutic resistance.

## Introduction

Gastrointestinal stromal tumors (GISTs) are rare mesenchymal neoplasms that arise in the gastrointestinal (GI) tract representing 3% of all tumors [1]. Even though GIST can arise anywhere, the stomach (60%) and small intestine (30%) are the most frequent locations. Duodenal GISTs are rare, accounting for 5% of all patients [2].

Advances in comprehending the fundamental molecular processes of GIST have resulted in the creation of logical, focused treatment strategies. Approximately 80% of GISTs have a mutation of the c-KIT or platelet-derived growth factor (PDGF) receptor [3]. In patients with unresectable, recurrent, or metastatic GIST, the median survival was 10-20 months [4]. After the introduction of imatinib, the overall response rates and progression-free survival (PFS) of the patients were high, with objective responses in over 50% of the patients [5]. Despite this success, pathological complete responses (pCR) to neoadjuvant imatinib remain uncommon. The indications and duration of treatment are less well-defined [6]. The question of whether surgery adds value over continuing on tyrosine kinase inhibitor (TKI) therapy alone is unanswered.

A rare case of pathological complete response after treatment with imatinib in a patient with recurrent duodenal gastrointestinal stromal tumor (GIST) is presented. This article was previously presented as a meeting abstract at the XLIII Congresso Nacional de Cirurgia on March 23, 2023.

## Case presentation

Medical history

A 66-year-old male was referred to our hospital due to a suspected recurrence of duodenal GIST. About his past medical history, he underwent surgery for a duodenal GIST five years before at another hospital, and an open local duodenal excision was performed. The histopathology showed a high-risk duodenal GIST, pT4, G1, N0, Ki67 5%, and mitotic count <5/50 high-power field (HPF). The resection margin was unaffected. After that, he undertook adjuvant treatment with imatinib 400 mg for 36 months and routine imaging surveillance. At the time of the referral, the patient was asymptomatic, denying abdominal distension, weight loss, or early satiety.

Investigation

**Figure 1:**
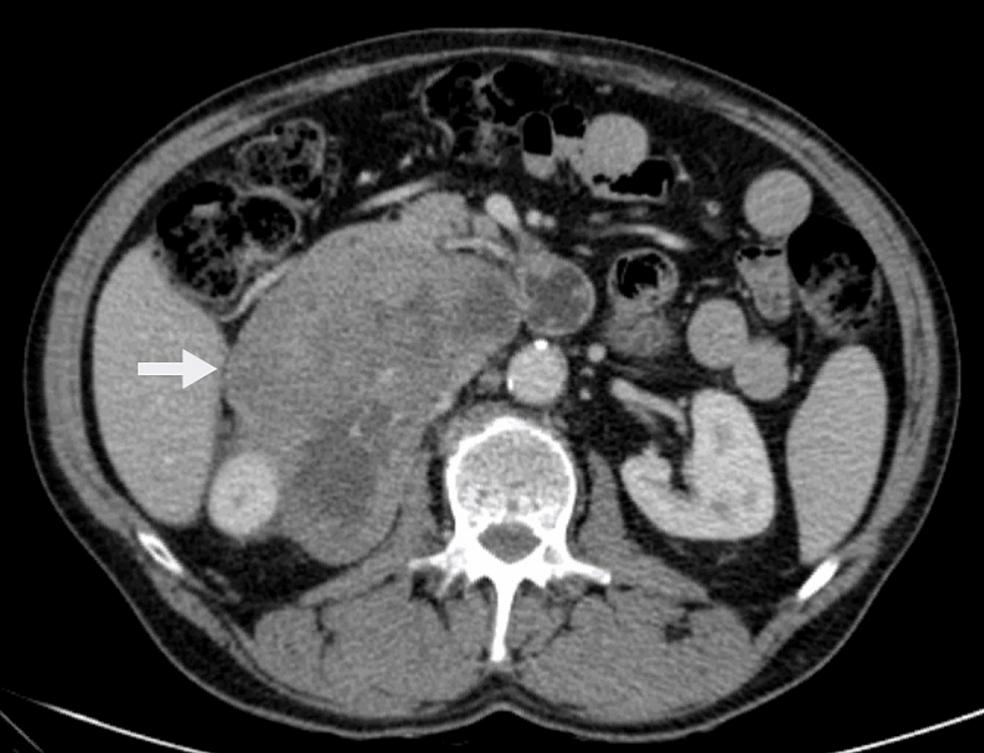
Local recurrence of GIST on the second duodenal portion on CT scan with an apparent invasion of the right kidney GIST, gastrointestinal stromal tumor; CT, computed tomography

Two years after the end of the adjuvant therapy, in a contrast-enhanced abdominopelvic computed tomography (CT) scan, a suspected local recurrence measuring 12 cm, with lobulated contours and heterogeneous density, in the second duodenal portion was identified with an apparent invasion of the right kidney and adrenal gland (Figure 1).

Upper gastrointestinal endoscopy did not show any changes other than a deformity in the transition between the first and second portions of the duodenum, which could be caused by a previous surgery or an extrinsic compression. An endoscopic ultrasound performed confirmed a sub-stenosis in the second duodenal portion as a consequence of an extrinsic heterogeneous lesion, hypoechoic with anechoic areas, with well-defined but irregular limits, reflecting a probable recurrence of the GIST.

The lesion was biopsied. Cytological analysis showed cell clusters with a spindle cell arrangement, without atypia or mitosis, compatible with GIST. The genetic study revealed a mutation in exon 11 of the imatinib-sensitive *KIT* gene.

Treatment

**Figure 2:**
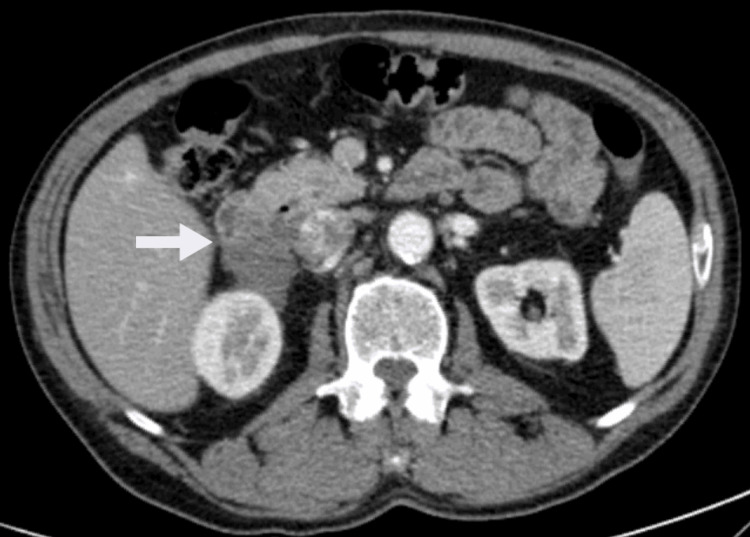
Reassessment CT scan after 12 months of neoadjuvant imatinib showing a decrease in tumor size CT: computed tomography

The patient was discussed in a multidisciplinary committee and proposed systemic therapy. Imatinib mesylate 400 mg per day was initiated. The treatment was well-tolerated, with no adverse events. After 12 months of therapy, a reassessment CT scan showed a decrease in tumor size to 5 cm (Figure 2).

The case was rediscussed by the multidisciplinary committee, and surgical intervention was decided. Given this, a cephalic duodenopancreatectomy was performed, which was uneventful. Intraoperatively, after an initial adhesiolysis, an extensive Kocher maneuver was done to perform a cephalic duodenopancreatectomy. The three anastomoses necessary for the reconstruction were performed the following way: a pancreaticojejunal anastomosis, according to the Heidelberg technique; an end-to-side hepaticojejunostomy; and a duodenojejunostomy. Care was taken to prevent tumor spillage and rupture. The final histopathology showed a pathological complete response with no residual neoplasia, despite the tumor's initial size. Adjuvant imatinib was restarted, and three years later, the patient had no evidence of recurrence or postoperative complications.

## Discussion

Duodenal GISTs account for 30% of all primary tumors of the duodenum [2]. Along with other GISTs, the size, anatomical location, mitotic index, and molecular behavior will determine the therapeutic possibilities and survival prognosis of these patients.

The treatment approach depends on the stage of the disease. In patients with localized and potentially resectable GIST, surgery is the primary treatment. R0 resection is still the only curative option for nonmetastatic tumors [7]. For patients with resectable disease but with significant predictable morbidity, neoadjuvant systemic therapy with TKIs should be chosen before surgery. The benefits of neoadjuvant imatinib have been demonstrated in large GISTs or GISTs in difficult locations (such as the esophagus or rectum) to facilitate organ-sparing surgery and reduce surgical morbidity [8,9]. The optimal duration of neoadjuvant TKIs is not established. The most consensual stated in the literature is TKI administration for four to 12 months since it was observed that with this period, the optimal response is typically obtained, the chance of secondary resistance developing is minimal, and the best outcomes can be obtained through surgery [10]. For unresectable or metastatic GIST, the cornerstone of the treatment is systemic therapy with surgery having a role merely in very selected cases.

In resectable disease, depending on the tumor size, location, proliferative activity, and intraoperative tumor rupture, half of all the patients may experience tumor recurrence after surgery. According to the literature, in the absence of adjuvant therapy, around 30% of the patients experience postoperative recurrence or metastasis within three years following resection [11]. Three years of imatinib is currently the standard adjuvant therapy for patients with a significant risk of recurrence [12].

Recurrence after complete resection should be managed as described for unresectable or metastatic disease. Recurrent disease represents locoregional metastases or the infiltrative spread of the tumor and has the same overall prognosis as distant metastases. In patients with metastasized GIST, surgery has been considered occasionally following neoadjuvant imatinib. The rationale behind combining systemic therapy and surgery is that a lower tumor load may reduce the likelihood of systemic therapy resistance [13]. By removing resistant clones, surgery aims to mitigate the spread of the tumor and eradicate it before a secondary resistance arises.

The knowledge of mutational analysis is required before starting systemic therapy. When compared to KIT exon 9 mutations or non-mutated KIT, the presence of a KIT exon 11 mutation is an independent predictor of longer progression-free survival (PFS) and overall survival (OS) in the patients treated with standard-dose imatinib in randomized studies [14].

The most effective way to evaluate tumor response is under debate. Fluorodeoxyglucose (FDG) PET-CT scanning is a highly sensitive method for the detection of GIST and other cancers with elevated glucose metabolism. Nevertheless, a routine eight to twelve weekly cross-sectional imaging of the abdomen and pelvis using a conventional CT scan is usually adequate. However, some GIST-responding tumors can increase in size during initial treatment as a result of intratumoral hemorrhage, necrosis, and degeneration [15].

It is unknown when patients with metastatic or recurrent GIST should undergo surgery. Several retrospective studies have shown a survival benefit of preoperative imatinib followed by surgery in patients with advanced or metastatic GIST who responded to preoperative imatinib [10]. The complete removal of residual metastatic disease is associated with a favorable prognosis. Whether this is due to surgery or patient selection bias for healthy patients has never been prospectively proven [16].

Only one small, multicenter, randomized controlled trial (RCT) compared surgery plus imatinib with imatinib alone to treat residual disease in patients with recurrent/metastatic GIST with peritoneal disease. In this trial, no statistically significant difference was observed between the two treatment regimens in two-year PFS, although OS was significantly longer in the surgery plus imatinib group [17].

Due to the lack of RCTs, the impact of surgery on PFS and OS in patients with recurrent or metastatic GIST remains unclear. In patients with no disease progression under systemic treatment and in whom a complete resection can be achieved, surgery should be considered.

The complexity of the duodenopancreatic region makes the surgical management of duodenal malignancies difficult. Because local lymph node involvement is rare in GISTs, the primary objective is to accomplish complete tumor resection with clear margins (R0 resection) without lymph node dissection. Surgical options range from local resection to pancreaticoduodenectomy, and the percentages of disease-free survival for the two surgeries are comparable [18]. Segmental or wedge resection can be performed in smaller tumors, located in the third or fourth duodenal portion, and is associated with a lower rate of complications. Pancreaticoduodenectomy is preferred for large GISTs with nearby structure invasion or involving the ampulla of Vater. Since this was a local recurrence and a segmental resection was not possible, the authors chose to perform a more extensive surgery [18].

Pathological complete response (pCR) after neoadjuvant imatinib is uncommon, ranging from 1% to 10% reported in case reports or small series [19]. Even in tumors nonviable in metabolic images, it usually contains viable cancer cells in pathological specimens [20]. It is currently unclear whether pCR rates can be further improved by extending the treatment duration.

Since our study is based solely on a case report, generalizing findings to a broader population is challenging, which diminishes the strength of the conclusions. Despite this, in the presented case, the early detection of recurrence and the initiation of systemic treatment may have had a positive impact on the results.

## Conclusions

In patients with recurrent duodenal GIST, systemic therapy with TKIs is the standard approach. The role of surgery in these cases is highly debated. Surgical approach is particularly indicated to patients who have favorable responses to imatinib after six to 12 months of treatment. In all cases, the surgical treatment must be individualized and agreed upon by a multidisciplinary committee.
